# Microplastics in feed cause sublethal changes in the intestinal microbiota and a non-specific immune response indicator of the freshwater crayfish *Procambarus clarkii* (Decapoda: Cambaridae)

**DOI:** 10.3389/fmicb.2023.1197312

**Published:** 2023-07-18

**Authors:** Rossy Guillén-Watson, Maria Arias-Andres, Keilor Rojas-Jimenez, Ingo S. Wehrtmann

**Affiliations:** ^1^Escuela de Biología, Universidad de Costa Rica, San José, Costa Rica; ^2^Escuela de Biología, Instituto Tecnológico de Costa Rica, Cartago, Costa Rica; ^3^Laboratorio ECOTOX, Instituto Regional de Estudios en Sustancias Tóxicas (IRET), Universidad Nacional, Heredia, Costa Rica; ^4^Centro de Investigación en Ciencias del Mar y Limnología (CIMAR), Universidad de Costa Rica, San José, Costa Rica; ^5^Centro de Investigación en Biodiversidad y Ecología Tropical (CIBET), Universidad de Costa Rica, San José, Costa Rica

**Keywords:** gut microbiota, crayfish, microplastics, freshwater, dysbiosis

## Abstract

Microplastics (MP) are a hazardous pollutant of global concern that threatens aquatic ecosystems and public health. We used the invasive, cosmopolitan, and environmentally versatile red swamp crayfish *Procambarus clarkii* as a model to study the effects of MP on the intestinal microbiome. Crayfish collected from the environment were compared with specimens exposed to recycled Polyethylene terephthalate (rPET) MP in feed (30%) for 96 h in the laboratory and a control group. We analyzed the 16S rRNA of the intestinal bacteria by PCR-DGGE and high-throughput sequencing. MP exposure caused dysbiosis of the intestinal microbiota, with an increase in Alphaproteobacteria and Actinobacteria. We detected higher abundance of opportunistic genera such as *Klebsiella*, *Acinetobacter*, *Hydromonas*, *Pseudomonas*, *Gemmobacter*, and *Enterobacter* on MP fed organisms. Moreover, MP exposure reduced the abundance of Clostridia and Bateroidetes, which are important for immune system development and pathogen prevention. Furthermore, MP exposure decreased the phenoloxidase (PO) immune response in crayfish. There was a significant difference in the richness of intestinal bacterial communities after consumption of food contaminated with MP, likely increasing the abundance of opportunistic bacteria in the intestinal microbiota. Our results suggest that MP alter the gut microbial composition and impair the health of *P. clarkii*.

## 1. Introduction

Microplastics (MP) are plastic fragments with a size of less than 5 mm (Cole et al., 2011). They are ubiquitous in freshwater ecosystems (Klein et al., 2015; Elizalde-Velázquez and Gómez-Oliván, 2021) and can be ingested by various aquatic organisms (Lechner et al., 2014; Eerkes-Medrano et al., 2015; Blettler et al., 2018; Scherer et al., 2018). The trophic transfer and bioaccumulation of MP from primary producers to consumers in freshwater food webs may result in adverse effects on the organisms at higher trophic levels (Mattsson et al., 2015). Moreover, MP can contain or adsorb additives, heavy metals, antibiotics, pesticides, and other environmental contaminants (Derraik, 2002). Furthermore, they can host beneficial or pathogenic microorganisms that form the “plastisphere” (Hirai et al., 2011; Zettler et al., 2013; Harrison et al., 2018; Amaral-Zettler et al., 2020; Liu et al., 2022). Therefore, the impacts of MP ingestion on the health of aquatic biota is a relevant research topic.

The transfer of microplastics (MP) along the food chains poses a potential risk for human health, as people may consume decapod crustaceans contaminated with MP (de Miranda and de Carvalho-Souza, 2016; D’Costa, 2022). Therefore, it is important to understand how commercial fish and shellfish are affected by MP and other environmental pollutants, as aquaculture is a growing source of global protein production (Zhou et al., 2021). A recent study detected MP fragments in all five decapod species sampled from Australian seafood markets and found that 48% of the specimens had MP pieces (Ogunola et al., 2022). However, most of the studies on MP exposure and effects in decapods focus on marine species (Devriese et al., 2015; Bordbar et al., 2018; Capparelli et al., 2022; Ogunola et al., 2022). Studies on MP presence on freswater decapods are very scarce, as mollusks and insects are relatively more represented in the literature of benthic freshwater invertebrates, as reviewed by D’Avignon et al. (2021). Therefore, additional studies focusing on freshwater decapods are needed.

The red swamp crayfish *Procambarus clarkii* (Girard, 1852) is the most cosmopolitan crayfish in natural environments, having adapted to different environments in more than 20 countries on all continents except Australia and Antarctica (Viccon-Pale et al., 2016; Loureiro et al., 2018). This crayfish has been recognized as the species with the greatest ecological plasticity of all decapods (Rodríguez et al., 2015). It was introduced in Costa Rica around 1966 and is now widespread in the country (Martín-Torrijos et al., 2021). This decapod species is economically and ecologically important, but little is known about its gut microbiota. Further, Pastorino et al. (2023) found this species as a good bioindicator of MP pollution in biotic and abiotic environment, in a study where Polypropylene (PP) and polyethylene terephthalate (PET) were the only MPs chemical types found. Still, few studies have examined the effects of microplastics (MP) on freshwater decapods like this species. The characterization of the digestive bacterial community in *P. clarkii* exposed to MP can provide insights into the additive toxicity of this pollutant (Shui et al., 2020; Capanni et al., 2021; Huang Y. et al., 2021; Wu et al., 2021; Xavier et al., 2021). Since this crayfish is a common food source for humans, the accumulation of MP and its impacts on the intestinal microbiota and immune response of *P. clarkii* are relevant for both ecosystem health and public health and aquaculture (D’Costa, 2022; Ogunola et al., 2022).

Experimental studies have shown that microplastics (MP) can cause oxidative stress, immunotoxicity, and reproductive and development toxicity in decapod crustaceans (D’Costa, 2022). These functions are closely related to the microbiome, especially in the gut (Diwan et al., 2022). The gut microbiota is the collection of microorganisms that live in the gastrointestinal tract and influence the health of their decapod host (Harris, 1993; Hsiao et al., 2013; Yano et al., 2015; Jin et al., 2018; Tang et al., 2021; Zhou et al., 2021). For example, Chae et al. (2019) showed how the microbiome can regulate the response to different pathogens in decapods (Chae et al., 2019; Foysal et al., 2021; Holt et al., 2021). Similarly, studies on the gut microbiota of the crayfish *P. clarkii*, revealed how bacterial communities interact and how dysbiosis can affect the crayfish health, ecosystem, and aquaculture productivity (Guo et al., 2020; Zhang et al., 2020; Zhang T. et al., 2021; Zhang et al., 2021a).

Environmental stress, such as changes in the aquaculture environment or contaminant input, can alter and ultimately destroy the microbial community in aquatic animals (Guo et al., 2020; Zhang et al., 2020; Huang Y. et al., 2021; Wu et al., 2021; Zhang T. et al., 2021; Zhang et al., 2021a), thus impairing host immunity. Changes in the gut microbial community of aquatic organisms, such as decapods, increase their risk of disease (Guo et al., 2020). Therefore, studies of the structure of gut microbiota help to assess the effects of contaminants on aquatic species. Plastic particles in particular, having a high surface-to-volume ratio, are colonized by microorganisms, including pathogens that can alter this structure, disrupting food webs, nutrient cycles, and the balance of aquatic ecosystems (Zettler et al., 2013; Kirstein et al., 2016; Arias-Andres et al., 2018).

More studies are needed to understand the effects of MP derived from commercially used plastics, as they are more abundant in the environment than virgin materials. Further, there is less ecotoxicological information from recycled resins compared to virgin materials. In this regard, PET is one of the most widely produced polymers in the world, and a major source of environmental pollution (Ranganathan et al., 2022). Recycling PET is a common practice to reduce waste and reuse this material for various applications, such as making new bottles or pavement construction (Sang et al., 2020; Enfrin et al., 2022). PET is the most recycled plastic with recycling rates of 31 and 52% in USA and Europe, respectively (Das et al., 2021). However, recycling processes can also generate microplastics (MP) from recycled PET (rPET), which can be released into the environment through effluents and sludge in dozens of mg/L and thousands of μg/g, respectively (Guo X. et al., 2022; Guo Y. et al., 2022). Moreover, rPET products can degrade and fragment under certain conditions, producing more MP (Rorrer et al., 2019; Yalcin-Enis et al., 2019; Abuaddous et al., 2021). Since most of the rPET in aquatic systems ends up in benthic habitats due to its density, it is important to assess the potential ingestion and effects of rPET MP in aquatic benthic animals such as decapod crustaceans.

The present study aimed to determine the sublethal effects of ingestion of recycled Polyethylene terephthalate, or rPET-MP, through food consumption. We used the invasive crayfish species *P. clarkii* as a model to assess the impact of MP ingestion on intestinal microbiota. The model species was selected due to its tolerance to a wide range of environmental conditions and its importance for human consumption (Tang et al., 2020). Consequently, the results obtained are relevant to both ecosystem health and human well-being. Changes in immune response were also measured as phenoloxidase (PO) activity in the hemolymph. PO is part of the innate defense system of invertebrates against pathogens and damaged tissues by melanization (Cerenius and Soderhall, 2004; Huang and Ren, 2020). The experimental setup allowed us to compare the gut microbiome of crayfish collected in the environment, where the exposure to MP may vary, to changes observed in the intestinal microbiota after a controlled MP exposure in the laboratory. This information is valuable for an ecological risk assessment of MP presence and sublethal effects in a commercially important freshwater decapod exposed to MP made from recycled weathered plastic.

## 2. Materials and methods

### 2.1. Obtention of individuals and gut tissue

We analyzed adult male individuals of the red crayfish *Procambarus clarkii* (Decapoda: Cambaridae), directly sampled from the environment, and after an exposure assay under laboratory conditions, with or without microplastics in the food. Three individuals of *P. clarkii* were collected in a reservoir formed by the Cachí Dam on the Reventazon River in Costa Rica (Coordinates: 9°49′42.8″N 83°48′45.3″W and 9°49′42.0″N 83°48′44.0″W) to analyze the gut microbiome under natural conditions. The site for collection was selected since it is a well-known habitat for the species, and the only location in Costa Rica where crayfish are sold for human consumption. The individuals were captured manually, using nitrile gloves and clean cloth bags, and then stored cold in coolers with ice until processing. Water temperature and pH were measured with a thermometer and pH strips at the sampling site. The identification of the red swamp crayfish was carried out following the morphometric and morphological characteristics described by Campos (2005). In addition, individuals obtained in the same reservoir by local vendors were purchased for the laboratory exposure assay. After collecting the crayfish from the environment, the specimens were weighed, measured, and dissected to obtain the intestine tube. Size (cm) of the crayfishes refers to total length, measured from tip of the rostrum to posterior median edge of the telson. The dorsal surface of each red swamp crayfish was washed with sterile water and disinfected with 70% v/v ethanol for 5 min. Subsequently, the digestive system was dissected with sterile surgical forceps and scissors. The intestine was removed by mechanical force (Meziti et al., 2010; Zhang et al., 2016). The gut sample was washed three times with sterile water and stored in sterile glass tubes at −80°C until DNA and MP extraction.

### 2.2. Production of feed with rPET-MP

Commercial plastic bottles of drinking water for human consumption and made entirely with rPET were manually cut using a razor blade and scissors before being blended using the laboratory knife mill GRINDOMIX gm300 (RETSCH) 15 times for 2 min at 3,500 rpm, followed by cooling for 5 min in a 4°C refrigerator, alternating blade direction each time. The resultant fragments were sieved through a stainless steel mesh to produce 0.5–1 mm particles. To make food containing 30% rPET, 150 g of rPET-MP was added to 500 g of powdered fish food (38% protein). A total of 50 g of cassava starch was then added, followed by 5 g of vitamin mix (ROVIMIX^®^ E50) and 500 mL of boiling water. The mixture was mixed by hand until a consistent texture was achieved and then pelletized and dried in an oven at 40°C for 24 h. Equivalent food size was achieved by blending with a food processor, sieving through a 4 mm screen, and retaining pellets captured on a 1 mm screen. The same process was carried out with pure pellets without MP.

To characterize the rPET particles, we performed Differential Scanning Calorimetry (DSC) using an SDT-Q600 thermal analyzer (TA Instruments, New Castle, DE) to detect and compare the characteristic endothermic reactions of pellets with rPET-MP and food pellets containing the rPET-MP. To perform the dynamic and isothermal analyses, we used 10 mg of each sample type. All DSC experiments were performed in a nitrogen atmosphere with a determined purge flow rate of 100 mL/min. The dynamic DSC was heated from room temperature to 800°C at a heating rate of 10°C/min (flow rate: 30 cm^3^/min). The temperature was monitored using a thermocouple inserted into the reactor to provide a graphical representation of the changes in sample mass as the temperature increased. Finally, the rate of change of sample mass as a function of temperature was plotted to simplify the weight reading as a function of the temperature thermogram peaks, which occur close together (Majewsky et al., 2016).

### 2.3. Laboratory exposure assay

Ten male specimens of *P. clarkii* were exposed to the control feed, and 10 were exposed to feed with MP. First, the specimens were acclimatized in the laboratory considering the parameters measured by Jin et al. (2018) and Zhang et al. (2020), adapting it to *P. clarkii* in the following conditions: The laboratory conditions were maintained at 26–28°C ± 2°C in fresh culture water (drinking water, filtered and sterilized with UV and well aerated; pH 7.6 ± 0.5) for 12 days for acclimatization. During the same period, the specimens were fed once a day every 48 h for 5 days with commercial food (Nutrafin commercial brand). Feed, droppings, and water changes were done every 48 h. Before laboratory experiments, the crayfishes were examined to ensure that they were of similar size and weight; subsequently, specimens were not fed for 48 h to empty their digestive systems.

Organisms were individually placed in 3 L glass recipients with 2 L of UV-treated water and 0.7 g of feed with or without MP. The specimens were kept at room temperature. Water and feed renewal were performed every 24 h for 96 h, and the following parameters were measured: pH, temperature (°C), dissolved oxygen (DO; mg/L), and conductivity (μS/cm). Subsequently, individuals were measured again, and their tissues were sampled as follows: (1) the specimens were placed at −20°C to numb them before hemolymph extraction using a sterile 1 mL/27-gauge syringe (JD-01 T2713-IB, NIPRO). A sample of 200 μL hemolymph was extracted dorsally from the base of the 5th walking leg and immediately placed in a microtube with 200 μL of EDTA-free anticoagulant pre-cooled at 4°C (Huang et al., 2010). From this hemolymph-anticoagulant mixture, 200 μL was centrifuged at 800 g for 10 min at 4°C. The plasma in the supernatant was quickly frozen with liquid N_2_ and stored at −80°C until enzymatic analysis. (2) Subsequently, individuals from treatment and control were decapitated and dissected to obtain half the gut for MP extraction and the other half for DNA extraction, as described before. Characterization of gut tissue and MP by electron microscopy, and microbiota analysis was performed for a subsample of five specimens from each treatment.

### 2.4. Phenoloxidase activity

The immunological response was assessed by measuring phenoloxidase activity (PO) in hemolymph based on Huang et al. (2010) and using L-DOPA solution as substrate (3 mg/mL in 0.1 M PPB buffer, pH 6.6). The plasma stored at −80°C was thawed, and 6 μL of hemolymph and 294 μL of L-DOPA solution was placed in triplicate on a 96-well spectrophotometer plate. The enzymatic reaction was measured at 490 nm every 10 s for a total time of 2 min. One unit of enzyme activity (U) was defined as a linear increase in absorbance of 0.001 per min. Total protein content (TP) in hemolymph was determined by the method of Bradford (1976), using bovine serum albumin (BSA) as standard. Enzymatic activity was normalized by TP (U/mg).

### 2.5. MP extraction from the gut

To extract MP, gut tissues were treated with 20 mL of 10% m/v KOH (Sigma Aldrich, St. Louis, Missouri, United States) for 3 weeks (Dehaut et al., 2016; Kühn et al., 2017). After digestion, the remaining solution was vacuum-filtered through 0.45 μm microfiber filter papers (47 mm diameter; Sartorius Stedim Biotech, Göttingen, Germany). Subsequently, filters were dried in an oven at 60°C for 48 h. A stereomicroscope (Leica Microsystem, Wetzlar, Germany) was used to inspect the MP particles collected from the intestinal tracts visually; MP were photographed and analyzed for color and shape. According to their shape, particles were classified into fibers (elongated) or fragments (angular and irregular pieces). In addition, to determine the presence of MP in the study samples, particles were separated by size, with a length of less than 2.5 mm, and stored in aluminum foil and glass Petri dishes for further microscopic analysis. A Hitachi High-Technologies TM3000 tabletop scanning electron microscope (SEM) with an accelerating voltage of 15 kV was used to observe the microstructure of the intestinal tissue of *P. clarkii* and associated MP. Cross-sections of the samples were placed on aluminum holders attached to a carbon sheet. Subsequently, we metalized the samples with gold–palladium to increase electrical conductivity using an EMS 150R ES ionic cover instrument (EMS, Hatfield, PA; Chae et al., 2019; Samal, 2020).

### 2.6. DNA extraction and sequencing

Five samples from each treatment in the bioassay were used for the DNA extraction, together with the three samples from specimens collected in the environment. Approximately 250 mg of the tissue was ground and extracted with the Power Soil Pro kit (Qiagen, United States) following the manufacturer instructions. The DNA samples from the intestine of *P. clarkii* were sent to Macrogen Corp (Beotkkot-ro Geumcheon-g, Seoul, Rep. South Korea) for 16S rRNA gene sequencing, targeting the V3-V4 region using the universal primers Bakt_341F: 5′-CCTACGGGGNGGCWGCAG-3′ and Bakt_805R: 5′-GACTACHVGGTATCTAATCC-3′, following the procedure of Klindworth et al. (2013). Sequencing was performed with the MiSeq sequencing platform (Illumina, San Diego, CA, United States).^1^ Libraries were prepared on a paired-end Illumina platform using the Nextera XT Index Kit V2 to generate 300 bp paired-end raw reads.

### 2.7. Bioinformatics

We used the DADA2 version 1.18 to process the Illumina-sequenced paired-end fastq files and to generate a table of amplicon sequence variants (ASVs), which are higher-resolution analogs of the traditional OTUs (Callahan et al., 2016). Briefly, we removed primers, inspected the quality profiles of the reads, filtered and trimmed sequences with a quality score < 30, estimated error rates, modeled and corrected amplicon errors, and inferred the sequence variants. After that, we merged the forward and reverse reads to obtain the full denoised sequences, removed chimeras, and constructed the ASV table. We assigned taxonomy to the ASVs with the function assignTaxonomy of DADA2, which uses as input the set of sequences to be classified and a training set of reference sequences with known taxonomy, which in this case was the SILVA database version 138 (Quast et al., 2013). We carried out an additional taxonomic assignment of the ASVs using the tool IDTAXA of DECIPHER (Murali et al., 2018) with the same version of SILVA and using the RDP database version 18.^2^ The consistency between the taxonomic assignments of the different programs and databases was verified followed by performing a manual curation. All sequences assigned to Eukaryota or Chloroplast were removed. The sequence data were deposited in the NCBI Sequence Read Archive under project PRJNA930915.^3^ This process generated 671.092 sequences from the 12 samples (mean length = 409 nt). The average number of sequences per sample was 55.924 (ranging from 49.089 to 64.961).

### 2.8. DNA amplification and DGGE

We performed a PCR in an AB applied biosystems thermocycler (Thermo Fisher Scientific, New York, United States) in 50 μL reaction volumes containing 0.3 mM of each primer, 0.2 mM of each dNTP (Thermo Fisher Scientific, New York, United States), and 0.03 U/μ Dream Taq DNA Polymerase (Thermo Fisher Scientific, New York, United States) as well as 1X of the Dream Taq Buffer, which contained KCl (NH_4_)_2_SO_4_ and 20 Mm MgCl_2_ (Thermo Fisher Scientific, New York, United States). The primers used for the PCR were 341F-GC (with GC clamp) (CCTACGGGAGGCAGCAGCGCCCGCCGCGCGCGGCGGGCGGGGCGGGGGCACGGGGGG) and 534R (ATTACCGCGGCTGCTGG) as a universal bacterial 16S rDNA reverse primer (Schäfer and Muyzer, 2001). The thermal cycling was as follows: initial denaturation at 94°C for 1 min, followed by 20 cycles of 94°C for 45 s, 65°C for 45 s (temperature decreases by 0.5°C with each new cycle, touchdown PCR-DGGE on the AB applied biosystems thermal cycler), and 72°C for 2 min. After that, another 20 cycles of denaturation at 94°C for 30 s were conducted, followed by annealing of 55°C for 30 s, extension at 72°C for 2 min, and one cycle for a final extension at 72°C for 10 min. The PCR products (expected sizes about 200 bp) were analyzed by running 5 μL aliquots of the reaction mixtures in 2% agarose (Merck, Darmstadt, Germany) gels.

The DGGE technique was performed using the Dcode Universal Mutation detection System (BIO-RAD, California, United States). We used 8% polyacrylamide gels (ratio of acrylamide and bisacrylamide 37:1) with a gradient of 45 to 65% denaturants (100% denaturant was defined as 7 M urea plus 40% formamide). The gels were run at 60°C (65 V) for 15 h in a 1X TAE buffer (40 mM Tris, 20 mM acetic acid, 1 mM EDTA, pH 8.3) and visualized with 1X GelRed^®^ Nucleic Acid Gel Stain (Biotium, California, United States). Then the gel staining was visualized using a UVISAVE HD2 transilluminator (Thermo Fisher, Diepoldsau, Switzerland). The bands obtained by DGGE were analyzed by clustering using the Unweighted Pair-Group Method Using an Arithmetic Average (UPGMA) method to construct molecular phylogenetic trees using the program Minitab^®^ 19.1.1 (Minitab, LLC, United States).^4^ The generated dendrogram was generated using Pearson’s correlation coefficient using the program Minitab^®^ 19.1.1 (Minitab, LLC, United States).

### 2.9. Statistical analysis

Statistical analyses and the visualization of results were performed with the R statistical program (R Core Team, 2019) and the Rstudio interface. Package Vegan v2.5–6 (Oksanen et al., 2020) was used to calculate alpha diversity estimators and non-metric multidimensional scaling analyses (NMDS). Data tables with the amplicon sequence variant (ASV) abundances were normalized into relative abundances and then converted into a Bray–Curtis similarity matrix. To determine if there were significant differences between the bacterial community compositions according to factors of (1) individuals obtained directly from the environment, as well as reared in laboratory conditions, exposed to feed (2) with or (3) without MPs we used the non-parametric multivariate analysis of variance (PERMANOVA) and pairwise PERMANOVA (adonis2 function with 999 permutations). In addition, we performed an Indicator Species Analysis to identify ASVs associated with a specific treatment, using Package Indicspecies version 1.7.9 with 999 permutations (De Cáceres et al., 2011). Phenoloxidase activity units were compared between control and treatment by nonparametric Kruskal-Wallis test.

## 3. Results

The males of *P. clarkii* collected from the Cachí reservoir and processed immediately had a smaller mean length of 4.36 ± 0.19 cm and mean weight of 19.56 ± 2.12 g compared to the 20 males used for the laboratory experiment. The control and exposed treatment specimens were of similar size with a mean initial length of 6.19 ± 0.60 cm, and mean initial weight of 29.11 ± 6.69 g (*t*-test *t* = 0.71894, df = 15.967, *p* = 0.4826; Supplementary Table S1). Crayfish-fed pellets with MP showed a slight increase in final weight relative to *P. clarkii* fed pellets without MPs (Supplementary Table S1; paired *t*-test, *p* = 0.013), but no differences in length were found either for control or MP treatment after 96 h. During field sampling, the water temperature in the reservoir was 21°C with a neutral pH. The water parameters measured during laboratory experiments at the beginning and end of media renewals were similar between the two treatments. In both cases, pH and dissolved oxygen (DO) decreased after 96 h, while temperature and conductivity increased (Supplementary Table S2). One organism died in each treatment during the exposure. Five organisms from the control had visible food in their intestines after 96 h, while the same was true for the MP treatment.

Phenoloxidase activity (normalized to TP) in hemolymph from control organisms was significantly higher (1.60 ± 1.06 U/mg TP) than the one measured in animals that had MP in their feed (0.28 ± 0.28 U/mg TP) (Kruskal-Wallis chi-squared = 5.63, df = 1, *p* = 0.017; Supplementary Table S5). Although mean TP in hemolymph was lower in animals fed with rPET (58.98 ± 16 mg/mL) than in those of control (73.26 ± 26 mg/mL), this difference was not statistically significant (Kruskal-Wallis chi-squared = 1.33, df = 1, *p* = 0.25).

DSC curves of rPET-MP and pellet mixture of food with the rPET-MP used for the bioassay (Supplementary Figure S1: red and black line, respectively) were measured to detect their characteristic endothermic reactions. The results showed two marked peaks in both samples: the first peak was at a melting temperature between 50 and 100°C and the second peak at a melting temperature of 250°C.

**Figure 1:**
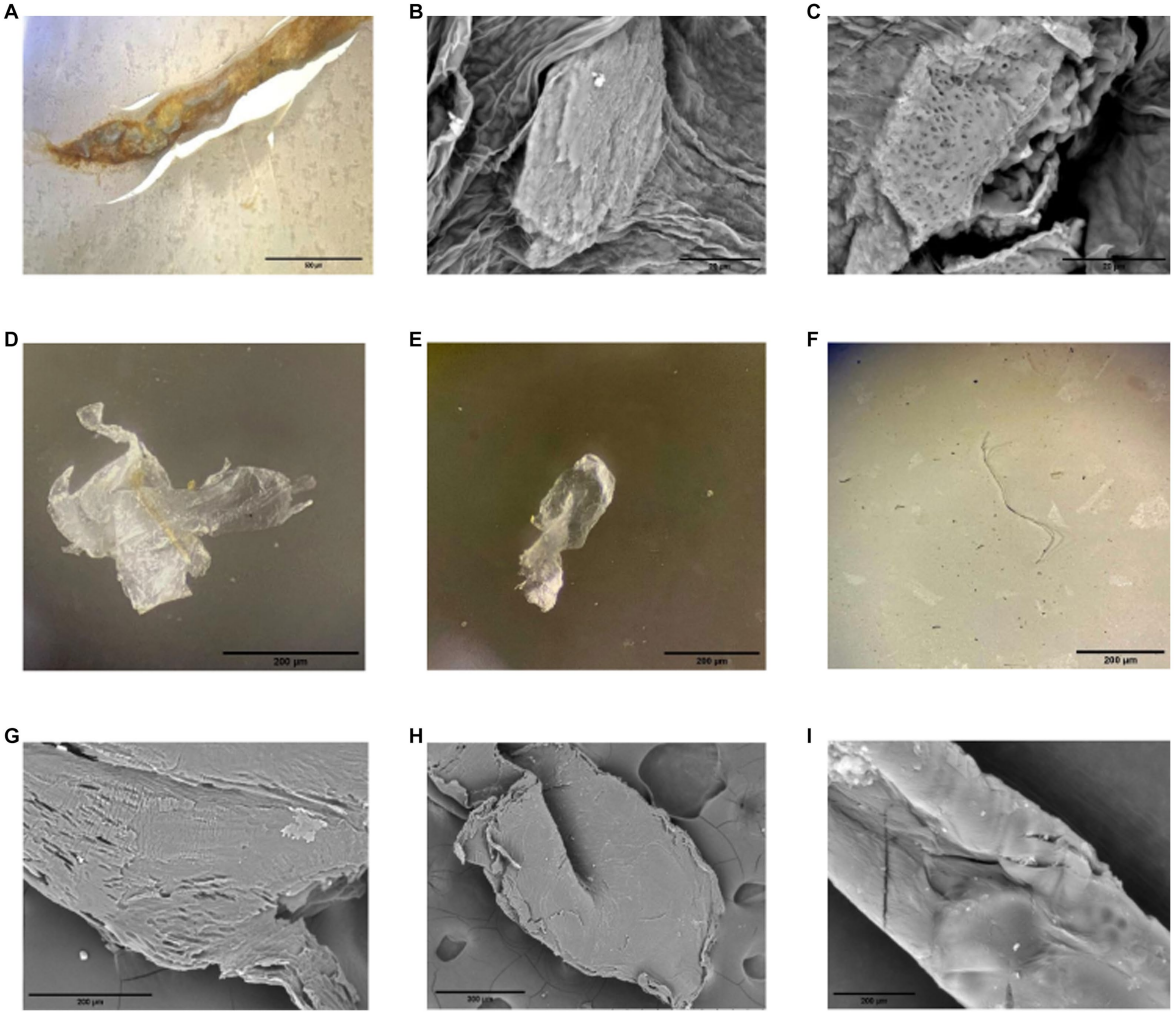
Microplastics (MP) found in gut samples of *Procambarus clarkii* fed with pellets with 30% rPET MP under light and SEM microscopes. MP can be seen from outside a section of the intestinal tube under the microscope **(A)** and inside the gut tissue in SEM images **(B,C)**. Forms included fragments **(D,E)** and fibers **(F)**. A closer look of particles **(D–F)** is presented on SEM images **(G–I)**, respectively, and shows irregularities within the different shapes of particles.

The presence of MP particles and fibers was observed in three out of five of the intestinal tracts of individuals fed pellets with MP (Supplementary Table S3; Figure 1). Particles embedded in tissue were observed by SEM (Figure 1). The irregular and porous nature of the particles obtained after the digestion of gut tissue can be seen in the SEM images (Figure 1). No MP-resembling residues were detected in the three control samples from the Cachí reservoir or in the four intestinal tracts of the individuals fed pellets without MP.

According to the analysis of sequences of the V3-V4 region of the 16S rRNA gene, the intestinal tract microbiota of *P. clarkii* comprised 1.053 amplicon sequence variants (ASVs). All the bacterial sequences were assigned to 19 phyla and 37 classes. Firmicutes was the most abundant group of phyla representing 45% of the sequences and 12% of the ASVs, whereas Proteobacteria comprised 40% of the sequences and 46% of the ASVs; Bacteroidota 13% of the sequences and 23% of the ASVs and Actinobacteria represented 0.7% of the sequences and 6.7% of the ASVs. Within Firmicutes, the most abundant genera were *Candidatus_Bacilloplasma*, *Candidatus*, *Hepatoplasma*, and *Erysipelothrix*. The Proteobacteria was dominated by *Citrobacter*, *Hafnia*, and *Shewanella*. *Bacteroides* were the most abundant genus within Bacteroidota and *Leucobacter* within Actinobacteroidota. No sequences of Archaea were detected in the intestinal tract of *P. clarkii*.

**Figure 2:**
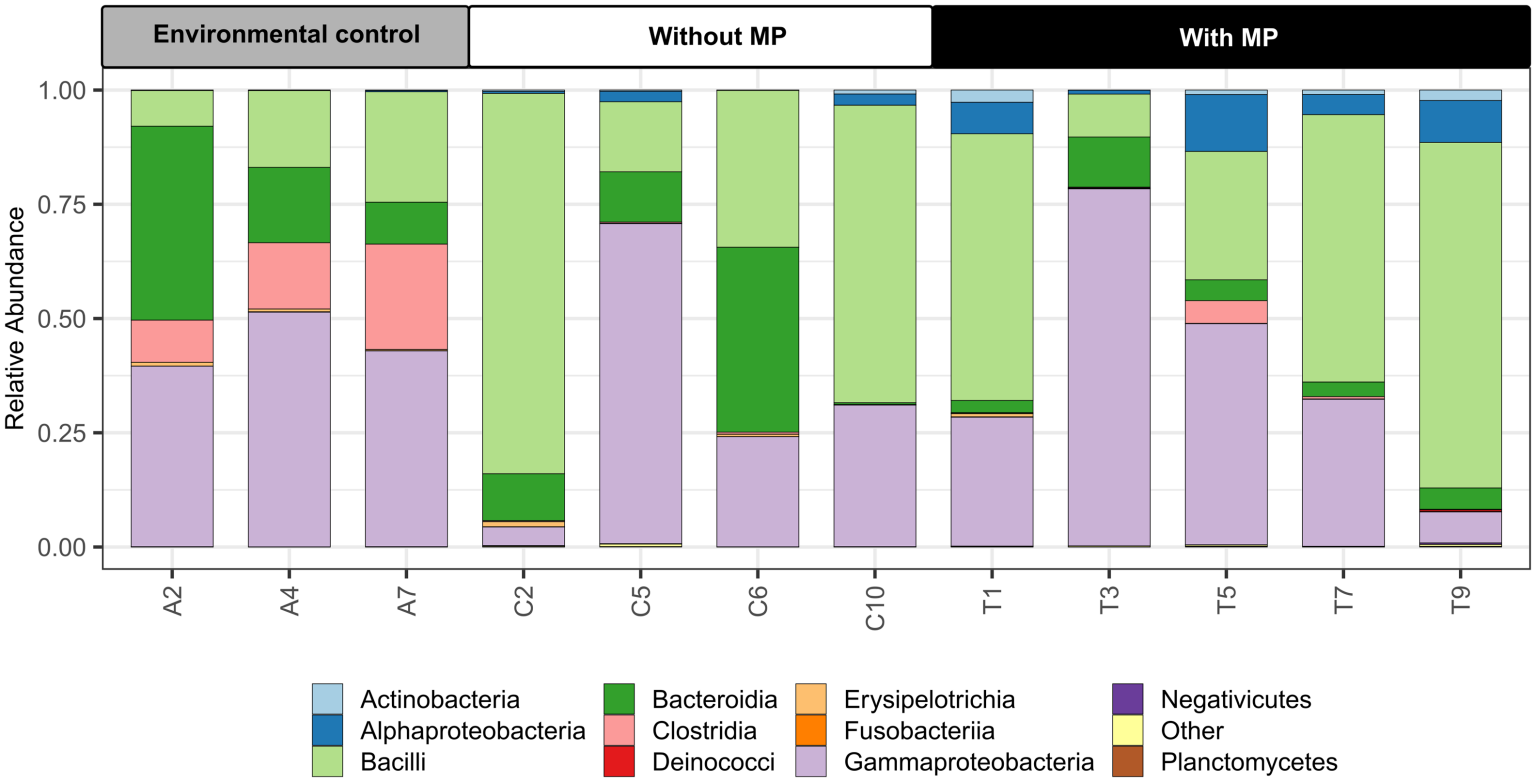
Relative abundances of dominant microbial communities at the class level in the intestine of *Procambarus clarkii* in environmental samples from the Cachí reservoir (A2, A4, and A7), treatment samples without microplastics (MP) (C2, C5, C6, and C10) and treatment samples with MP (T1, T3, T5, T7, and T9).

Some differences between the treatments analyzed were determined at the class level (Figure 2). Guts studied from individuals from the Cachí reservoir presented a higher abundance of Gammaproteobacteria, Clostridia, and Bacteroidia compared to individuals maintained in the laboratory. According to the indicator species analysis, particularly the genus *Tyzzerella* (Clostridia) represented an indicator species of the digestive tract of the guts from the control Cachí reservoir specimens. Samples from crayfishes of the laboratory experiment (with and without MP) had a lower abundance of Clostridia. The intestinal tracts from crayfishes fed pellets with MP contained a higher proportion of Alphaproteobacteria and Actinobacteria than the control specimens from the Cachí reservoir and fed pellets without MP. According to the indicator species analysis, the predominant genera in the gut microbiota of specimens fed pellets with MP were: *Klebsiella*, *Acinetobacter*, *Hydromonas*, *Pseudomonas*, *Gemmobacter*, and *Enterobacter*. Also, a significant decrease of bacteria belonging to the Bacteroidia was observed in gut samples of specimens fed pellets with MP.

**Figure 3:**
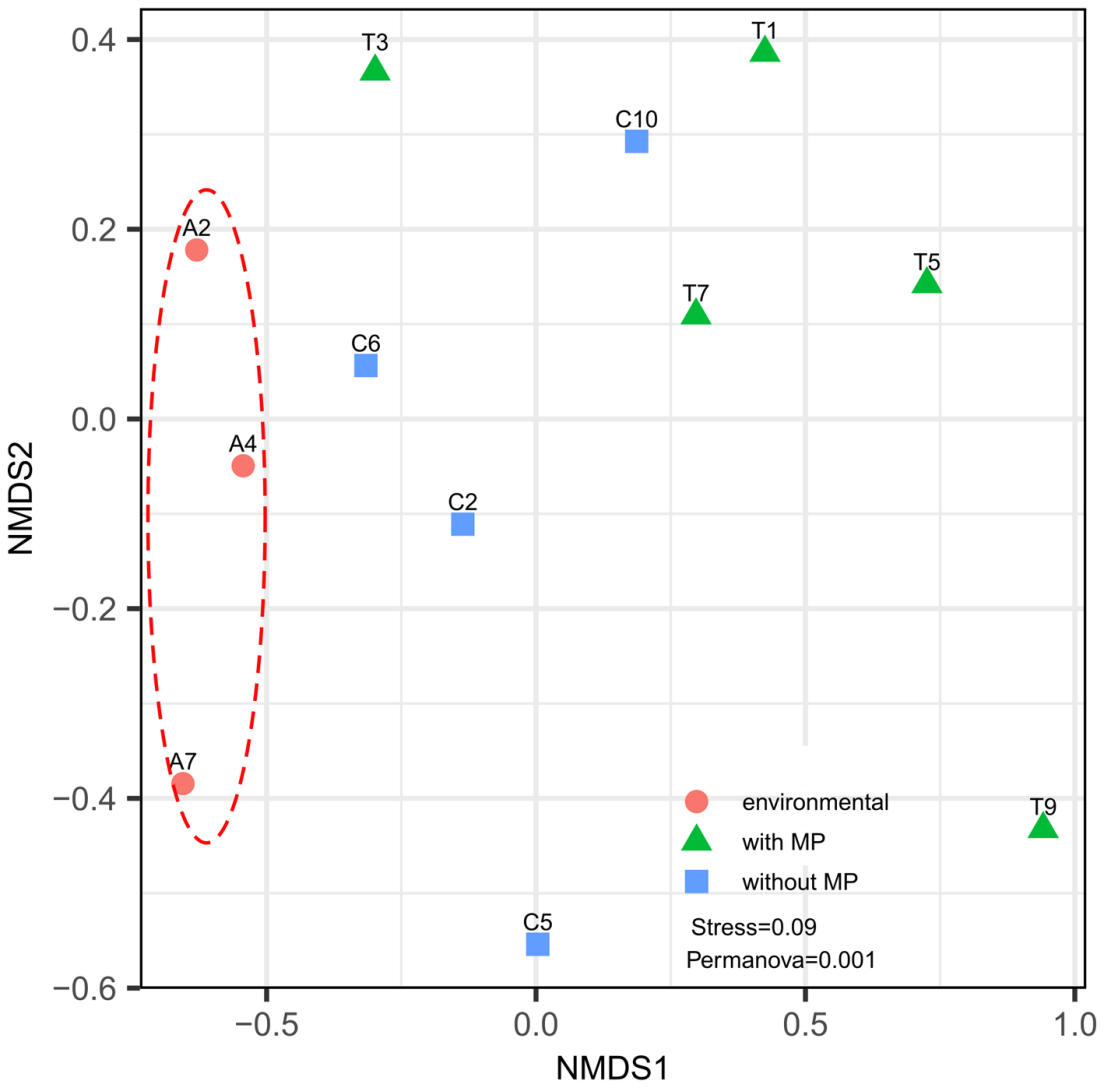
Non-metric multidimensional scale (NMDS) reflecting the degree of variation in intestinal bacterial communities of *Procambarus clarkii*. Data points of the same color represent the same sample type. Different symbols denote gut samples from crayfishes in control Cachí reservoir (circles) and after laboratory treatments (with MP as triangles and without MP as squares). Analysis reveals that gut microbiome in *P. clarkii* showed significant variation, but clusters according to treatment.

The NMDS analysis of the bacterial community composition showed that samples from the different treatments were separated from each other (Figure 3). The structure of the bacterial communities of the intestinal tract from specimens taken directly from the Cachí reservoir separated clearly from the communities of the intestinal tracts of the red swamp crayfishes from laboratory experiments. The samples from *P. clarkii* fed with pellets with and without MP are generally separated except for some samples that overlap. However, according to the statistical analysis, differences between the structure of the communities per treatment were significant (Permanova, *p* = 0.034).

When analyzing the alpha diversity estimators, individuals fed pellets with feed containing MP presented an average richness of 238 ASVs in their microbiota compared to 156 ASVs in individuals fed pellets without MP and 81 ASVs in the gut samples of *P. clarkii* from the Cachí reservoir (Supplementary Figure S2A). According to the Kruskal-Wallis test, these differences were statistically significant (*p* = 0.028). The Shannon diversity index values of the gut samples of individuals fed pellets with MP were slightly higher (average of 3.01) compared to 2.4 and 2.7 of the intestinal samples from red swamp crayfishes fed pellets without MP and specimens from the Cachí reservoir, respectively. These differences, however, were not significantly different (Kruskal-Wallis test: *p* > 0.5) (Supplementary Figure S2B, see test details in SI).

DNA banding patterns amplified satisfactorily between 190 and 200 bp with 25 bands discernible for each sample (Supplementary Figure S3; Supplementary Table S4). Some bands matched samples from both the Cachí reservoir and samples from the experimental conditions, while other bands were found only in samples taken from experimental conditions. The samples from individuals fed pellets with MP showed a decrease in the intensity of six common bands (Supplementary Figure S3: bands 5, 6, 9, 9, 11, and 12) with respect to the control samples from the Cachí reservoir and organisms fed with pellets without MP. Four unique bands could be identified in individuals fed pellets with MP (Supplementary Figure S3: bands 18, 19, 20, and 21). In contrast, in the red swamp crayfishes fed with pellets without MP only one unique band was identified (Supplementary Figure S3: band 24). On the other hand, there were two common bands in samples obtained from the pellet-fed individuals with and without MP (Supplementary Figure S3: bands 10 and 22), and other two common bands in the gut samples of the specimens fed without MP and those collected from the Cachí reservoir (Supplementary Figure S3: bands 8 and 25). There was only one common band (Supplementary Figure S3: band 7) in the red swamp crayfishes pellet-fed with MP and those from the Cachí reservoir. This band profile is presented as a matrix in Supplementary Information (Supplementary Table S4) and was used for a cluster analysis (Supplementary Figure S4) that highlights the division in two groups: Group 1 (from water sample, control organisms from the reservoir, four samples from organisms not fed with MP, and four from organisms fed pellets with MP) and Group 2 (sediment sample, one control not fed with MP and one fed with MP). The results of the cluster analysis revealed that the first group was more genetically diverse than the second one.

## 4. Discussion

Our results showed that the intake of weathered MP fragments from recycled PET in feed altered the bacterial communities in the intestinal tract of *P. clarkii* after 96 h exposure. This finding is significant because it shows the effects of recycled polymer MP mixed with food. The microbiota is crucial for the intestinal health of crayfishes, as it facilitates nutrient absorption and stimulates immune responses and disease resistance in hosts (Zhang et al., 2020). Furthermore, the immune response of crayfishes fed with rPET-MP, measured by phenoloxidase activity in plasma, differed from that of non-rPET controls. These results underscore the importance of studying complex and multifactorial biological indicators of MP effects, such as changes in the microbiome and its relationship to host health.

Previous studies have highlighted the importance of investigating sub-lethal effects using MP that reflect environmental materials and applying better characterization methods during bioassays (Guo X. et al., 2022; Guo Y. et al., 2022). PET is a major source of MP production, and its recycled form (rPET), as used in this study, is of interest and debate for upcycling and circular economies, making it relevant for current and future environmental exposure. Our microscopic analysis revealed that the rPET-MP used in this study were fiber-shaped or amorphous, clear-colored fragments less than 2 mm, after weathering by grinding (original bottles were blue). This size range of MP was similar to that found in commercial feed in proportions like the nominal percentage used in our study (Wang et al., 2022). Moreover, the presence of MP in the food pellets was confirmed by a DSC thermal analysis. The rPET-MP showed a characteristic endothermic peak around 250°C that matched the maximum melting temperatures reported for PET (Majewsky et al., 2016). This signal was also detected in the food pellets with MP prepared for our experiment. The first marked peak in the pellets with MP (from around 50 to 100°C) could be due to protein denaturation, as proteins decompose at a temperature between 50 and 60°C (Ahmed et al., 2021), and the pellets contained 38% protein source in their composition.

MP was exclusively extracted from the guts of individuals fed with pellets with plastics. This finding agrees with previous observations that MP can accumulate in the internal tissues of crayfish, posing a potential health risk to aquatic animals and humans consuming crayfish (Capanni et al., 2021; Zhang T. et al., 2021; Zhang et al., 2021a). Unexpectedly, no MP were found in the samples directly taken from the Cachí reservoir, even with the high level of pollution known in this watershed (Mora Alvarado, 1997). A plausible explanation is that organisms had time to excrete MP particles before analysis. Moreover, smaller particles (nm-μm range) might have escaped our extraction procedure.

Water parameters during the laboratory exposure remained within the acceptable range for the growth of *P. clarkii*. Exposure of the intestinal microbiome to the surrounding water occurs in early developmental stages of crustaceans during oviposition, but environmental conditions and diet are particularly relevant in the definition of intestinal microbiomes of crustaceans (Xie et al., 2021). In our study, the PCR-DGGE as well as high-throughput sequencing of the 16S rRNA showed that shifting conditions from the reservoir to the laboratory provoked changes of the crayfish gut microbiota. However, both analyses were consistent and revealed higher bacterial richness in the gut of organisms fed pellets with MP compared to the control without MP. In this regard, there are reports of similar dysbiosis results associated to a response in the immune system (Clerissi et al., 2020; Tan et al., 2021; Wu et al., 2021). When the host becomes sick, dysbiosis in the gut microbiota occurs together with physiological adjustments in digestion, metabolism, and immunity (Wu et al., 2021). Therefore, consuming feeding pellets containing rPET MP generates in *P. clarkii* similar symptoms as observed in sick organisms.

Studies where a transient impact of MP on crustacean immunity was observed, suggest plastic polymers produce an activation of such system with the purpose to return to homeostasis, which is not always elicited by natural particles, and that can be different from that of co-exposed chemicals (Dolar et al., 2021, 2022). Although it is recognized that there is still not enough assessments on interactions of MP in general with immune systems, and even less for specific polymers (Yang et al., 2022), hypothesis could be tested regarding rPET in relation to the characteristics of the polymer or the chemicals that co-occur with it. For example, packaging materials made from recycled PET contain heavy metal catalysts like antimony (Sb) (Whitt et al., 2016), which can be released from PET bottles (Filella, 2020) and influence the gut microbial community of other invertebrates (Huang B. et al., 2021).

The phenoloxidase (PO) activity of crayfishes fed pellets with MP in our experiment had a different reaction than the organisms from the control treatment. PO activity in shellfish is a standard measure of invertebrate immunity and response to microbial pathogens (Coates and Söderhäll, 2021). An increase in PO activity is usually associated with stress and disease, while decreased PO activity has been related to depleted proteins and immune-compromised animals (Coates and Söderhäll, 2021). Several studies demonstrated that low oxygen and low pH as well as chemical pollutants in water could result in decreased PO activity (Tanner et al., 2006; Coates and Söderhäll, 2021). A decrease in PO activity tied with changes in the gut microbiota of crustaceans has been linked with exposure to environmental pollutants such as pesticides (Fu et al., 2022) and, more recently, ingestion of μm-sized, pure polyethylene and polystyrene MP (Liu et al., 2019; Zhang et al., 2022). Adaptation of symbiotic bacteria in support of the homeostasis of the host is partly due to the immune system control by PO (Hooper et al., 2012; Groussin et al., 2020; Wu et al., 2021). Our results support that rPET MP ingestion prompts both an immune response and dysbiosis; however, the description of how these reactions develop in time during MP ingestion remains to be evaluated with more detail.

High-throughput sequencing analysis of 16S rRNA from the 12 intestinal tracts showed that, overall, Firmicutes, Proteobacteria, Bacteroidota, and Actinobacteria were the predominant phyla in the intestinal microbiota of *P. clarkii*, which is in agreement with other studies conducted with the same species (Guo et al., 2020; Zhang et al., 2020; Wu et al., 2021; Xie et al., 2021). These four phyla play principal roles in intestinal functions of digestion, absorption, and immunity of aquatic decapods such as *P. clarkii* (Li et al., 2020; Duan et al., 2021). The most abundant genera encountered in our study of Firmicutes were *Candidatus*_*Bacilloplasma*, *Candidatus,*_*Hepatoplasma*, *Erysipelothrix*, and *Candidatus*. These genera also constitute the core microbiota of the crayfish species *Cherax cainii*, and are known to play an essential role in crayfish digestion and immunity (Wei et al., 2019; Shui et al., 2020; Foysal et al., 2021).

Among Proteobacteria, the most abundant genera were *Citrobacter*, *Hafnia*, and *Shewanella*, coinciding with the analysis of gut microbiota in *P. clarkii* (see Wu et al., 2021). These genera include opportunistic pathogenic bacteria in some freshwater decapods (Zhang et al., 2020; Wu et al., 2021; Zhang T. et al., 2021; Zhang et al., 2021a), but their abundance may also be attributed to the presence of foreign compounds in the diet or external environment (Zhang et al., 2016; Foysal et al., 2021). Such changes may cause an alteration in the stability of the gut microbiota of *P. clarkii*, and thus may facilitate or hinder infection by pathogenic bacteria in the crayfish (Zhang et al., 2020; Foysal et al., 2021). Finally, *Bacteroides* was the most abundant genus within Bacteroidota, and *Leucobacter* within Actinobacteria. In this regard, Wu et al. (2021) concluded that a predominance of *Leucobacter* might indicate possible dysbiosis and the presence of diseases in *P. clarkii*.

The gut content of specimens collected in the Cachí reservoir was predominated by classes of Gammaproteobacteria, Bacteroidia (Bacteroidetes), and Clostridia (Firmicutes). In dams, such as Cachí, the denitrification process depends on heterotrophic and autotrophic bacteria, and the abundance of Gammaproteobacteria and Bacteroidia is high (Zhang et al., 2021b). Also, Bacteroides microorganisms are known to produce carbohydrate metabolism-related enzymes and to promote food digestion in the human gut (Karlsson et al., 2011). The increase of this genus in the gut of *P. clarkii* has been interpreted as an adaptation strategy after exposure to xenobiotics (Zhang T. et al., 2021; Zhang et al., 2021a). Meanwhile, species of Clostridia are crucial in the modulation of physiological, metabolic, and immunological processes in the gut by interacting with other resident microbial populations (Lopetuso et al., 2013; Setälä et al., 2014). Finally, our analyses of the gut content revealed the presence of the genus *Tyzzerella* exclusively in specimens collected from the Cachí reservoir, which has an abundant aquatic vegetation. This finding agrees with the results of Shui et al. (2020), who reported this genus as frequent in *P. clarkii* specimens reared in rice fields. This bacterial genus could be related to the capacity of strains to metabolize plant polysaccharides and a broad feeding spectrum, including herbivorous items in *P. clarkii* (see Shui et al., 2020).

The intestinal tract of the specimens fed with pellets that contained MP showed a higher proportion of Alphaproteobacteria and Actinobacteria compared to those from control Cachí reservoir and fed pellets without MPs samples. Alphaproteobacteria are known to include a variety of aromatic hydrocarbon-degrading strains (Ghosal et al., 2016), while Actinobacteria produce metabolites with antimicrobial activity and are involved in the degradation of recalcitrant compounds (Ranjani et al., 2016). Also, Actinobacteria participate in the decomposition of organic matter present in sediments (Shui et al., 2020), and play a crucial role in maintaining intestinal homeostasis in humans (Glenny et al., 2017; Wang et al., 2021) and possibly also in crustaceans, with some strains applied as probiotics (Das et al., 2008; Li et al., 2018; Gainza and Romero, 2020). An increase of Alphaproteobacteria and Actinobacteria, already predominant groups in the gut content of *P. clarkiii*, was also found by Zhang T. et al. (2021) and Zhang et al. (2021a) after exposure to the anti-inflammatory drug Diclofenac (mg/L). Finally, Duan et al. (2021) and Wang et al. (2021) observed an increase in these two phyla after 5 μm sized MP exposure (μg/L-mg/L) in the marine shrimp *Litopenaeus vannamei*, without feeding and after 48 h. Therefore, our results are congruent to other studies indicating these two phyla are involved in the response of crustaceans to emergent contaminants.

The most abundant genera in our samples of organisms fed with pellets containing MP were: *Klebsiella*, *Acinetobacter*, *Hydromonas*, *Pseudomonas*, *Gemmobacter*, and *Enterobacter*. Although these genera can be part of the intestinal microbiota of healthy humans, some strains are of clinical importance (Vogel et al., 2012; Hadder et al., 2018). Moreover, species of *Klebsiella*, *Acinetobacter*, *Pseudomonas*, and *Enterobacter* (KAPE) are of particular concern due to their ongoing acquisition of genetic traits such as antibiotic resistance and virulence (Boucher et al., 2009). In this regard, Eckert et al. (2018) showed that MP promote the persistence of typical indicators of microbial anthropogenic pollution in natural waters. However, additional research is needed to demonstrate the transfer of human pathogenic bacteria to crayfish through MP.

The most abundant genera in gut samples of MP-fed crayfishes also comprise potential biodegrader species of recalcitrant xenobiotics (Vaz-Moreira et al., 2015; Liu et al., 2020), and further studies need to explore biodegradation metabolism of gut microbiome after exposure to MP. Additionally, the decrease of Bacteroides levels observed in MP-fed crayfishes suggests that MP exposure affects putative beneficial bacteria linked to the degradation of lignin and environmental pollutants in the gut of *P. clarkiii*, as seen in *L. vannamei* exposed to MP (Duan et al., 2021).

Clostridia is another class of bacteria that suffered a considerable decrease in gut samples of specimens fed with pellets containing MP. Generally, these microorganisms play a crucial role in developing the immune system, modulating immune tolerance, and helping to prevent the establishment of potentially harmful and pathogenic organisms (Kelly et al., 2005; Lopetuso et al., 2013). Therefore, this observed decrease in our crayfish specimens could suggest a reduction in the resistance to pathogen colonization and a possible dysbiosis effect in the presence of MP (Kelly et al., 2005).

We found a dysbiosis effect in the intestinal tract of *P. clarkii* with molecular tools after feeding them with rPET-MP in an irregular form. This result was associated with changes in the biochemical immune response of the crayfish PO. Since previous studies have shown that gut microbiome differs between male and female of *P. clarkii*, affecting their immune responses (Chen et al., 2022), we suggest including females in future studies to examine the toxic effects of MP on crustaceans, by assessing histological and physiological changes as well as hemolymph microbiome reactions in the presence of MP. Studying other markers of crayfish immune system such as cellular responses (e.g., hemocyte parameters), receptors and signaling molecules (Bouallegui, 2021) would be useful to fully understand the relationship between the animal’s physiological response and microbiota changes.

The characteristics of the plastic used in our experiment and the exposure by feeding are more environmentally relevant than usual exposure to virgin materials mixed in water. However, further chemical characterization of recycled resins, and potential associated toxic substance release during processing of the resin, are necessary to understand the cause-effect relationship behind their effect on biota, as we cannot discriminate to the precise mechanism of actions involved at cellular level. Also, we recognize the importance of comparing the responses of other recycled resins that have proven high toxicity as virgin materials such as Polystyrene or Polyvinyl chloride. Finally, we recommend studying sub-lethal MP effects along with microbiome changes to enhance the production and safer consumption of crustacean species in aquaculture and provide input for the conservation of species in natural ecosystems.

## Data availability statement

The datasets presented in this study can be found in the NCBI Sequence Read Archive under accession number PRJNA930915: https://www.ncbi.nlm.nih.gov/sra/PRJNA930915 and in the article and the Supplementary material.

## Ethics statement

Ethical review and approval was not required for the study of animals in accordance with the local legislation and institutional requirements.

## Author contributions

RG-W and MA-A contributed equally to conception, design of the study, data interpretation, and writing the first draft of manuscript. KR-J contributed to data interpretation and statistical analysis. IW contributed to the design of the study and data interpretation. All authors contributed to manuscript revision, read, and approved the submitted version.

## Funding

This study was partially financed by the Consejo Nacional de Rectores (CONARE), Costa Rica, and inscribed at the Vicerrectoría de Investigación of the Universidad de Costa Rica (# 808-C0-656 and 808-B8-297 with IW as principal investigator), and inscribed at Universidad Nacional (SIA 0643-19). Publication process was financed by Universidad Nacional.

## Conflict of interest

The authors declare that the research was conducted in the absence of any commercial or financial relationships that could be construed as a potential conflict of interest.

## Publisher’s note

All claims expressed in this article are solely those of the authors and do not necessarily represent those of their affiliated organizations, or those of the publisher, the editors and the reviewers. Any product that may be evaluated in this article, or claim that may be made by its manufacturer, is not guaranteed or endorsed by the publisher.

## Footnotes

^1^ https://dna.macrogen.com/

^2^ http://rdp.cme.msu.edu/

^3^ https://www.ncbi.nlm.nih.gov/sra/PRJNA930915

^4^ https://www.minitab.com/en-us/products/minitab/free-trial/
