# Supplementary material for: Microplastics in feed cause sublethal changes in the intestinal microbiota and a non-specific immune response indicator of the freshwater crayfish Procambarus clarkii (Decapoda: Cambaridae)
Source: Front Microbiol. 2023 Jul 18;14:1197312. doi: 10.3389/fmicb.2023.1197312 (PMC10390773; doi:10.3389/fmicb.2023.1197312)
Supplement: Supplementary file 1 [file Data_Sheet_1.docx]

Supplementary Material

Microplastics in feed cause sublethal changes in the intestinal microbiota and a non-specific immune response of the freshwater crayfish *Procambarus clarkii* (Decapoda: Cambaridae)

**Rossy Guillén-Watson, Maria Arias-Andres^*^, Keilor Rojas-Jimenez, Ingo S. Wehrtmann**

*** Correspondence:** Corresponding Author: maria.arias.andres@una.cr

# Supplementary Figures and Tables

## Supplementary Figures


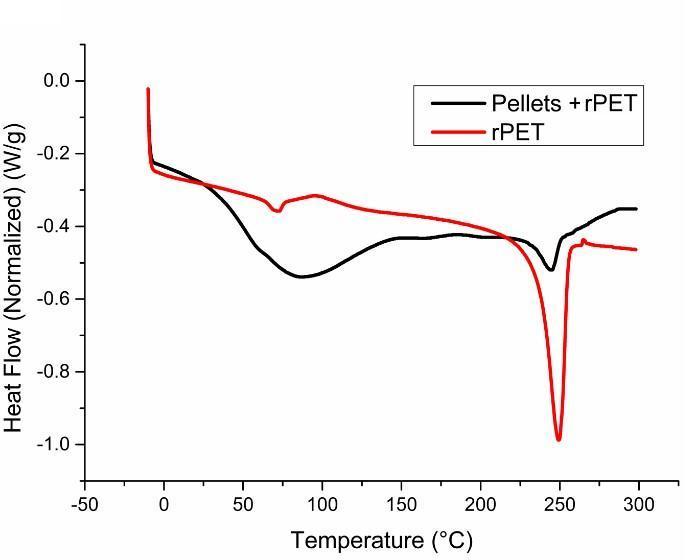


**Supplementary Figure 1.** Differential Scanning Calorimetry (DSC) signals from food pellets with 30% rPET MPs (black line), and only rPET-MPs (red line).


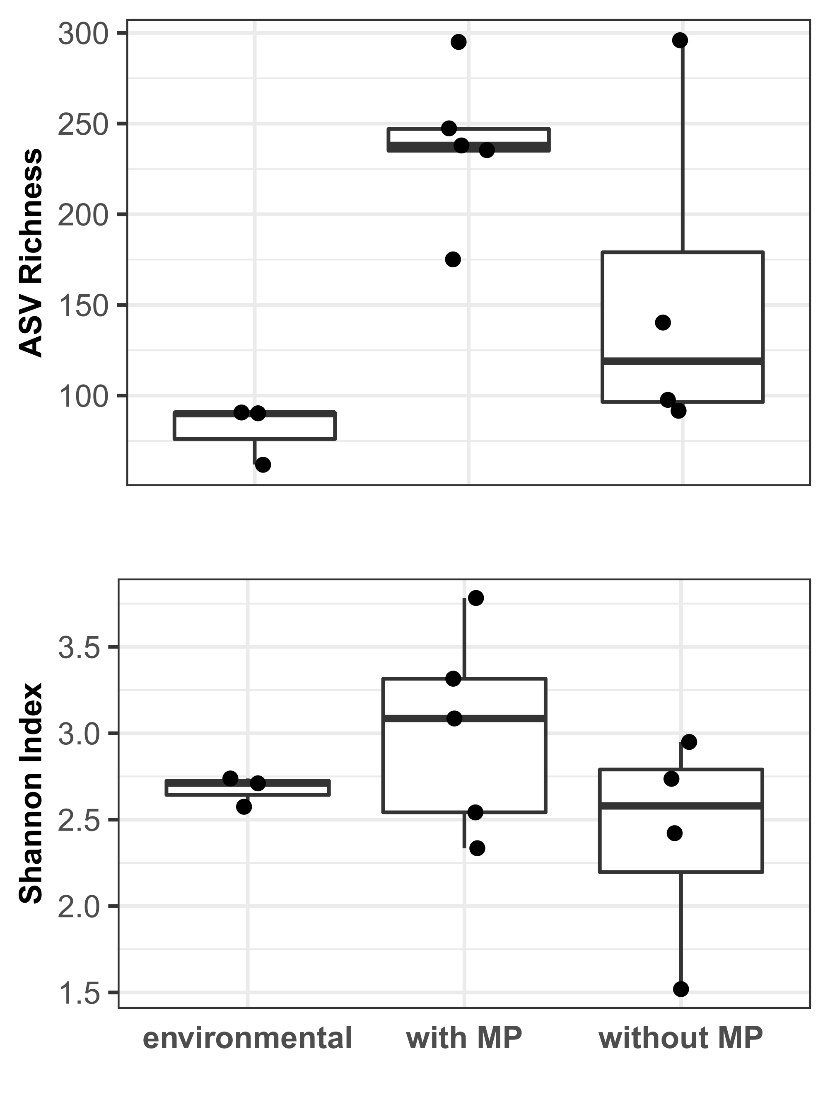


**Supplementary Figure 2.** Alpha- and Beta-diversity of intestinal microbial communities of *Procambarus clarkii* among environmental samples from the Cachí reservoir, Costa Rica, and the exposure groups with and without MPs. (A) ASV (amplicon sequence variant) for species richness, these different are statistically significant according to the Kruskal-Wallis test (p = 0.028); (B) Shannon index for species diversity with an average of 3.01, compared to 2.4 and 2.7 of the samples without microplastics and from the Cachí reservoir, respectively.


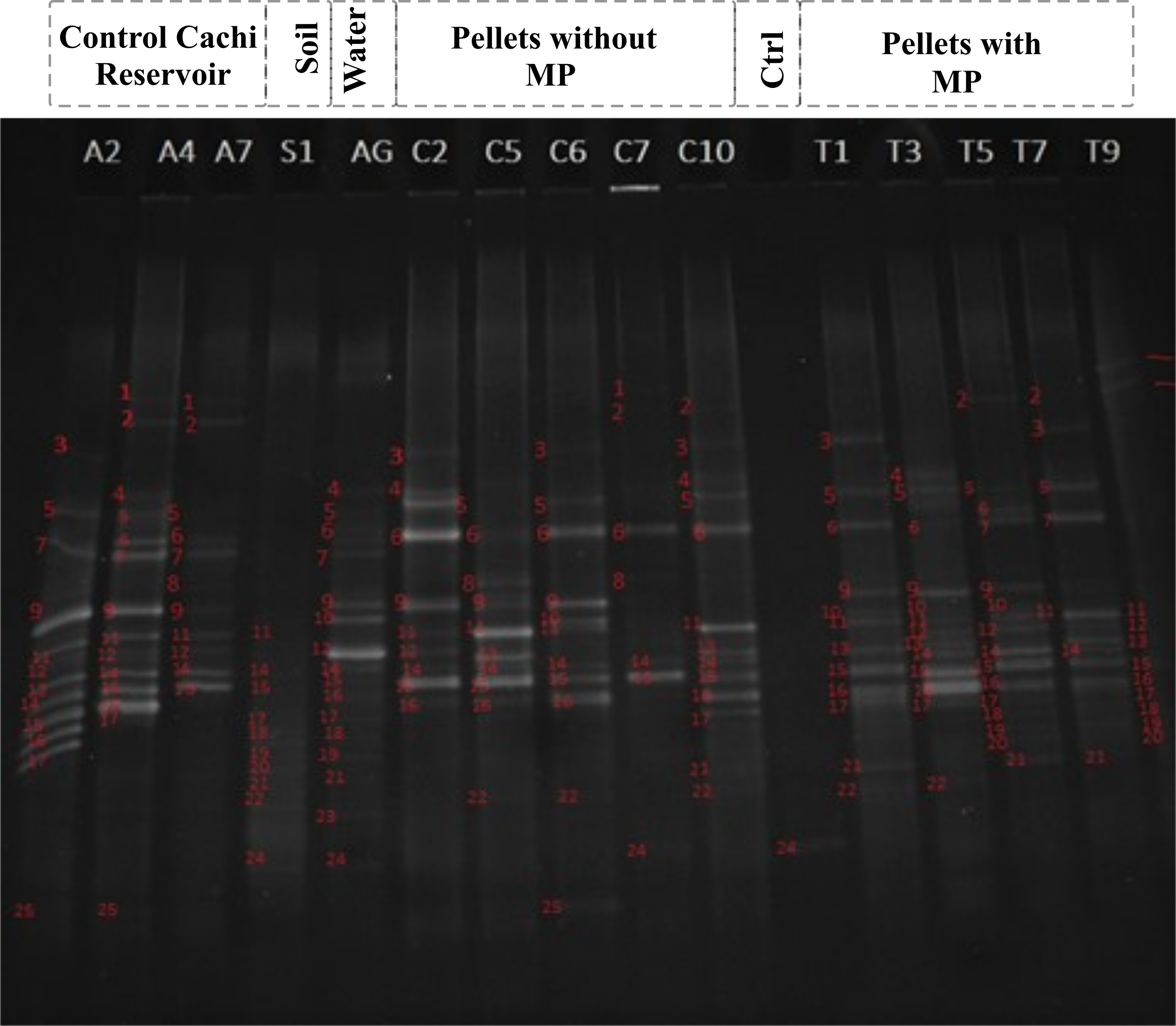


**Supplementary Figure 3.** Results of PCR-DGGE from the red swamp crayfish *Procambarus clarkii* guts. A= Control from the Cachí reservoir, Costa Rica; S= Soil, AG= Water, C= fed pellets without microplastic and T= fed pellets with microplastic. A total of 25 different bands were counted in all samples analyzed.


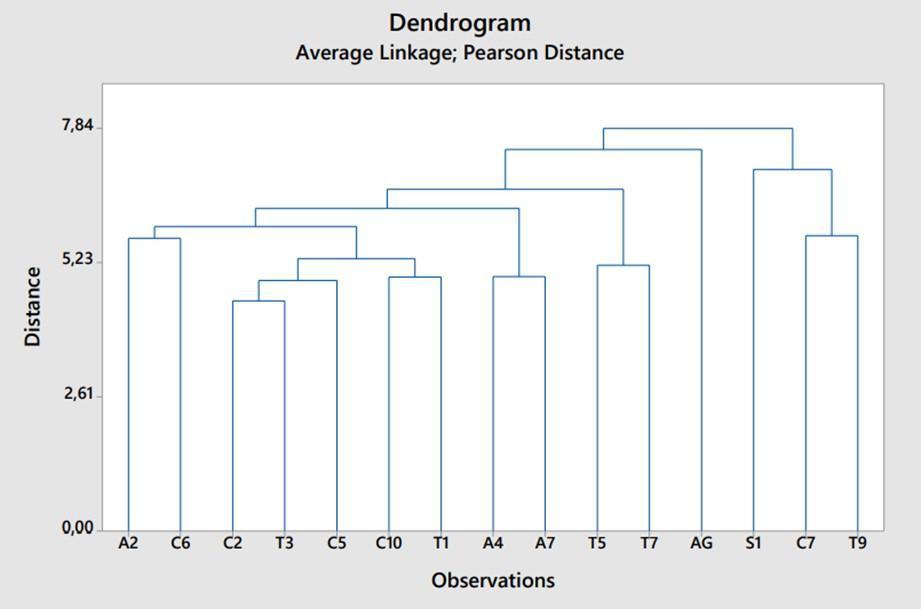


**Supplementary Figure 4.** 16S rRNA dendrogram of the intestinal tracts of the red swamp crayfish *Procambarus clarkii* and water and soil samples from the collection site. * A= Control Cachí reservoir, Costa Rica; S= Soil, AG= Water, C= fed pellets without microplastic and T= fed pellets with microplastic. Data generated from Pearson's correlation coefficient, performed with Minitab® 19.1.1 statistical software (Minitab, LLC, United States).

## Supplementary Tables

## Supplementary Table 1. Measurements of adult *Procambarus clarkii* reared under laboratory conditions.

| **Description** | **Minimum** | **Maximum** | **Standard**  **Average deviation** | |
| --- | --- | --- | --- | --- |
| **Total length (cm)**  measured from tip of the rostrum to posterior median edge of the telson |  | | | |
| Initial treatment with pellets without MPs | 5.61 | 7.45 | 6.28 | 0.62 |
| Final treatment with pellets without MPs | 5.81 | 7.56 | 6.39 | 0.61 |
| Initial treatment with pellets with MPs | 5.31 | 7.30 | 6.09 | 0.60 |
| Final treatment with pellets with MPs | 5.86 | 7.14 | 6.58 | 0.46 |
| **Weight (g)** |  | | | |
| Initial treatment with pellets without MPs | 18.87 | 39.16 | 28.59 | 6.69 |
| Final treatment with pellets without MPs | 22.82 | 39.33 | 30.27 | 6.10 |
| Initial treatment with pellets with MPs | 25.21 | 34.84 | 29.62 | 3.09 |
| Final treatment with pellets with MPs | 19.07 | 39.33 | 29.99 | 5.39 |

**Supplementary Table 2.** Measured water parameters of adult *Procambarus clarkii* reared under experimental conditions for 96 hrs.

| **Description** | **pH** | | **Temperature (°C)** | | **DO (mg/L)** | | **Conductivity (µS/cm)** | |
| --- | --- | --- | --- | --- | --- | --- | --- | --- |
| **Treatment with pellets without MPs** | **Initial** | **Final** | **Initial** | **Final** | **Initial** | **Final** | **Initial** | **Final** |
| Minimum | 7.17 | 6.30 | 20.70 | 20.90 | 7.96 | 6.51 | 253 | 275 |
| Maximum | 7.17 | 7.15 | 20.70 | 21.40 | 7.96 | 7.87 | 253 | 282 |
| Average | 7.17 | 6.58 | 20.70 | 21.18 | 7.96 | 7.43 | 253 | 279.50 |
| Standard deviation | 0.0 | 0.39 | 0.00 | 0.22 | 0.00 | 0.63 | 0.00 | 3.11 |
|  | | | | | | | | |
| **Treatment with pellets with MPs** | **Initial** | **Final** | **Initial** | **Final** | **Initial** | **Final** | **Initial** | **Final** |
| Minimum | 7.17 | 6.28 | 20.70 | 21.20 | 7.96 | 7.38 | 253 | 270 |
| Maximum | 7.17 | 6.45 | 20.70 | 21.80 | 7.96 | 7.86 | 253 | 280 |
| Average | 7.17 | 6.35 | 20.70 | 21.52 | 7.96 | 7.59 | 253 | 275.60 |
| Standard deviation | 0.00 | 0.06 | 0.00 | 0.26 | 0.00 | 0.18 | 0.00 | 4.16 |

**Supplementary Table 3.** Microplastics (MPs) identified in samples of intestinal tracts of *Procambarus clarkii* maintained in the laboratory and fed with pellets containing MPs.

| **Sample** | **MP Types** | | **Color** |
| --- | --- | --- | --- |
|  | **No. Fibers** | **No. fragments** |  |
| T1 | 1 | 6 | Transparent |
| T3 | 2 | 1 | Transparent |
| T7 | 2 | 0 | Black |

**Supplementary Table 4.** Profile matrix of 16S RNA banding of 13 intestinal tract samples of *Procambarus clarkii* for banding pattern analysis, based on the presence and absence of bands.

|  | **Samples** | | | | | | | | | | | | | | |
| --- | --- | --- | --- | --- | --- | --- | --- | --- | --- | --- | --- | --- | --- | --- | --- |
| **Band** | **A2** | **A4** | **A7** | **S1** | **AG** | **C2** | **C5** | **C6** | **C7** | **C10** | **T1** | **T3** | **T5** | **T7** | **T9** |
| 1 | 0 | 1 | 1 | 0 | 0 | 0 | 0 | 0 | 1 | 0 | 0 | 0 | 0 | 0 | 1 |
| 2 | 0 | 1 | 1 | 0 | 0 | 0 | 0 | 0 | 1 | 1 | 0 | 0 | 1 | 1 | 1 |
| 3 | 1 | 0 | 0 | 0 | 0 | 1 | 0 | 1 | 0 | 1 | 1 | 0 | 0 | 1 | 0 |
| 4 | 0 | 1 | 0 | 0 | 1 | 1 | 0 | 0 | 0 | 1 | 0 | 1 | 0 | 0 | 0 |
| 5 | 1 | 1 | 1 | 0 | 1 | 1 | 1 | 1 | 0 | 1 | 1 | 1 | 1 | 1 | 0 |
| 6 | 0 | 1 | 1 | 0 | 1 | 1 | 1 | 1 | 1 | 1 | 1 | 1 | 1 | 0 | 0 |
| 7 | 1 | 1 | 1 | 0 | 1 | 0 | 0 | 0 | 0 | 0 | 0 | 0 | 1 | 1 | 0 |
| 8 | 0 | 0 | 1 | 0 | 0 | 0 | 1 | 0 | 1 | 0 | 0 | 0 | 0 | 0 | 0 |
| 9 | 1 | 1 | 1 | 0 | 1 | 1 | 1 | 1 | 0 | 0 | 1 | 1 | 1 | 0 | 0 |
| 10 | 0 | 0 | 0 | 0 | 1 | 0 | 0 | 1 | 0 | 0 | 1 | 1 | 1 | 0 | 0 |
| 11 | 1 | 1 | 1 | 1 | 0 | 1 | 1 | 1 | 0 | 1 | 1 | 1 | 1 | 1 | 0 |
| 12 | 1 | 1 | 1 | 0 | 1 | 1 | 0 | 0 | 0 | 0 | 0 | 1 | 1 | 1 | 0 |
| 13 | 1 | 0 | 0 | 0 | 0 | 0 | 1 | 0 | 0 | 1 | 1 | 1 | 0 | 1 | 0 |
| 14 | 1 | 1 | 1 | 1 | 1 | 1 | 1 | 1 | 1 | 1 | 0 | 1 | 1 | 1 | 0 |
| 15 | 1 | 1 | 1 | 1 | 1 | 1 | 1 | 1 | 1 | 1 | 1 | 1 | 1 | 1 | 1 |
| 16 | 1 | 1 | 0 | 0 | 1 | 1 | 1 | 1 | 0 | 1 | 1 | 1 | 1 | 1 | 0 |
| 17 | 1 | 1 | 0 | 1 | 1 | 0 | 0 | 0 | 0 | 1 | 1 | 1 | 1 | 1 | 0 |
| 18 | 0 | 0 | 0 | 1 | 1 | 0 | 0 | 0 | 0 | 0 | 0 | 0 | 1 | 1 | 0 |
| 19 | 0 | 0 | 0 | 1 | 1 | 0 | 0 | 0 | 0 | 0 | 0 | 0 | 1 | 1 | 0 |
| 20 | 0 | 0 | 0 | 1 | 0 | 0 | 0 | 0 | 0 | 0 | 0 | 0 | 1 | 1 | 1 |
| 21 | 0 | 0 | 0 | 1 | 1 | 0 | 0 | 0 | 0 | 1 | 1 | 0 | 1 | 1 | 1 |
| 22 | 0 | 0 | 0 | 1 | 0 | 0 | 1 | 0 | 0 | 1 | 1 | 1 | 0 | 0 | 0 |
| 23 | 0 | 0 | 0 | 0 | 1 | 0 | 0 | 1 | 0 | 0 | 0 | 0 | 0 | 0 | 0 |
| 24 | 0 | 0 | 0 | 1 | 1 | 0 | 0 | 0 | 1 | 0 | 0 | 0 | 0 | 0 | 0 |
| 25 | 1 | 1 | 0 | 0 | 0 | 0 | 0 | 1 | 0 | 0 | 0 | 0 | 0 | 0 | 0 |

* 1 means presence of the band and 0 absence of the band. Red numbers show a possible change in bacterial community structure when one or more of the bands are absent in one sample but present in another type of sample.

**Supplementary Table 5.** Measured protein and phenoloxidase activities in the hemolymph of *Procambarus clarkii* individuals reared under experimental conditions for 96 hrs.

| **Treatment** | **Replicate** | **Sample** | **Protein mg/mL** | **U/mL** | **U/mg of protein** |
| --- | --- | --- | --- | --- | --- |
| Control | 1 | C1 | 62,2438103 | 137,73406 | 2,21281536 |
| Control | 2 | C2 | 125,313364 | 193,044412 | 1,54049341 |
| Control | 3 | C4 | 38,3835931 | 104,032937 | 2,71034909 |
| Control | 4 | C5 | 71,4133441 | 187,917827 | 2,63141055 |
| Control | 5 | C6 | 70,2961614 | 181,140866 | 2,57682443 |
| Control | 6 | C7 | 62,6863658 | 37,7292133 | 0,60187272 |
| Control | 7 | C8 | 75,4611077 | 137,183868 | 1,81794135 |
| Control | 8 | C9 | 52,2107536 | 15,0196434 | 0,28767337 |
| Control | 9 | C10 | 101,291237 | 0 | 0 |
| MP | 1 | T1 | 69,5729609 | 0 | 0 |
| MP | 2 | T2 | 80,5397018 | 24,9123779 | 0,30931798 |
| MP | 3 | T3 | 72,1041625 | 20,515427 | 0,28452486 |
| MP | 4 | T5 | 51,9624907 | 34,983611 | 0,67324739 |
| MP | 5 | T6 | 30,7899885 | 0 | 0 |
| MP | 6 | T7 | 57,1922013 | 38,4644407 | 0,67254695 |
| MP | 7 | T9 | 62,2977805 | 16,3030754 | 0,26169593 |
| MP | 8 | T10 | 47,4182014 | 0 | 0 |
